# A Single-Center Review of Renal Replacement Therapy in Patients With Acute Traumatic Brain Injury

**DOI:** 10.7759/cureus.100279

**Published:** 2025-12-28

**Authors:** Gena Topper, Michael Bamimore, T Hess, Jacob Metheny, Patrick Morris, Jared Plumb, Amber Valeri, Krystal Hunter, Corey Mossop, Tanya Egodage

**Affiliations:** 1 Department of General Surgery, Cooper University Hospital, Camden, USA; 2 Department of Neurological Surgery, Cooper University Hospital, Camden, USA; 3 Department of Trauma, Cooper Medical School of Rowan University, Camden, USA; 4 Department of Neurological Surgery, Tower Health Medical Group, West Reading, USA; 5 Department of Trauma, Cooper University Hospital, Camden, USA

**Keywords:** acute kidney injury, intermittent hemodialysis, renal replacement therapy, trauma, traumatic brain injury

## Abstract

Background: Traumatic brain injury (TBI) is a major cause of morbidity, and concomitant acute kidney injury presents substantial management challenges. Continuous renal replacement therapy (CRRT) is thought to cause less hemodynamic instability than intermittent hemodialysis (IHD) after TBI, but factors associated with modality selection in TBI remain unclear. We sought to describe the clinical characteristics and outcomes of patients with TBI requiring renal replacement therapy and to test whether injury severity, Glasgow Coma Scale (GCS), and comorbidity burden were associated with the receipt of CRRT vs. IHD.

Methods: This 10-year retrospective study at a level I trauma center included adult TBI patients requiring renal replacement therapy. Patients were grouped by modality (CRRT vs. IHD), and demographics, injury severity, and outcome variables were compared.

Results: Eighty-one patients met the inclusion criteria; 52 received IHD and 29 received CRRT. Patients receiving IHD were older (69 vs. 58 years, p=0.006) and had higher Charlson Comorbidity Index scores (6.6 vs. 3.5, p<0.001). Patients receiving CRRT had lower presenting GCS (8.7 vs. 13.5, p<0.001) and higher injury severity scores (24.5 vs. 16.2, p=0.006). CRRT patients had higher mortality (62.1% vs. 30.8%, p<0.001), longer hospital stays (17 vs. 8.6 days, p=0.001), and were more commonly discharged with severe disability (17.2% vs. 3.8%, p=0.001).

Conclusion: In this retrospective cohort, we found that CRRT was more frequently used in TBI patients with worse injuries and lower levels of consciousness on presentation, whereas patients with greater comorbid burden were more likely to receive IHD. These findings highlight the need for evidence-based guidelines to inform renal replacement modality selection in acute TBI.

## Introduction

Traumatic brain injury (TBI) is associated with significant morbidity and mortality in the United States, with incidence continuing to rise nationally. Acute kidney injury (AKI) occurs in approximately 9-20% of patients with TBI [1-4]. Moore et al. reported a 9.2% incidence of AKI in moderate to severe TBI [1], while Robba et al. identified a 12% incidence of AKI of any severity within the first week after injury [2]. AKI in this population is associated with prolonged ICU admission, increased mortality, and worse long-term neurologic outcomes [2]. These patterns vary with the severity of TBI and concomitant injuries, the level of care at the admitting hospital, and the length of hospitalization [1-6]. A subset of TBI patients also present with pre-existing chronic kidney disease (CKD) or end-stage renal disease (ESRD), further complicating management and necessitating a better understanding of appropriate renal replacement strategies when acute and/or chronic renal dysfunction are present, as chronic dysfunction can introduce additional physiological derangements in the setting of critical illness [7,8]. As the population ages, there has been renewed interest in the epidemiology and management of renal dysfunction in this subgroup [2,5,9]. However, the ideal management of patients with TBI and concomitant renal dysfunction requiring continuous renal replacement therapy (CRRT) remains unknown.

Intermittent hemodialysis (IHD) is commonly used for ESRD and acute renal failure but may worsen intracranial edema and potential herniation in TBI patients due to dialysis disequilibrium syndrome (DDS) [10-12]. DDS can manifest as nausea, vomiting, headache, seizures, or coma following early dialysis treatments, and results from significant osmotic gradients and more rapid clearance of urea from the blood than the brain, both promoting cerebral edema. This phenomenon can be dangerous in outpatient IHD patients, and potentially fatal in patients with severe TBI, particularly those with a depressed level of consciousness. CRRT has been shown to minimize intracranial volume shifts; therefore, it has been recommended as an alternative for patients at higher risk of developing DDS, such as those with a TBI [13-15]. Davenport, among others, has recommended that CRRT be used for patients with TBI and intracranial hypertension who require renal replacement therapy to decrease the risk of DDS [16,17]. While CRRT offers more hemodynamic stability and reduced risk of intracranial volume shifts and subsequent DDS compared with IHD, standardized recommendations for modality selection of renal replacement therapy in acute TBI patients remain absent from current Brain Trauma Foundation guidelines [18-20].

Given these gaps, further characterization of how and why renal replacement modalities are selected in TBI patients is needed. The objective of this study was to describe the clinical characteristics and outcomes of TBI patients requiring renal replacement therapy at a single, urban level I trauma center and to test whether injury severity, presenting Glasgow Coma Scale (GCS), and comorbidity burden were associated with the use of CRRT vs. IHD. This framework reflects the exploratory nature of the study and avoids implying causality.

## Materials and methods

This is a 10-year retrospective observational trial conducted at a single urban level I trauma center using the Institutional Trauma Registry. It is compliant with the Strengthening the Reporting of Observational Studies in Epidemiology criteria and received approval from the local institutional review board. Patients were captured by querying the institutional trauma registry. Inclusion criteria consisted of adult patients (≥18 years old) admitted to the ICU with TBI on admission, who required renal replacement therapy in the posttraumatic period. TBI was captured by the presence of a head Abbreviated Injury Score and confirmed by computed tomography (CT) scan. The query was performed for patients admitted between January 1, 2010, and December 31, 2020. Patients who sustained isolated concussions or calvarial fractures without concomitant intracranial hemorrhage were excluded from the study, as were patients whose records were missing any of the collected data points, as described below.

​Patient demographics, comorbidities, clinical parameters, and outcomes were captured, including age, sex, admission GCS, and injury severity score (ISS). Charlson Comorbidity Index (CCI, calculated using manual chart review), type of intracranial hemorrhage on CT scan as reported by an independent neuroradiologist blinded to patient outcomes and the type of renal replacement therapy used, and the type of renal replacement therapy used (IHD vs. CRRT) were noted. Outcomes included hospital length of stay (HLOS), intensive care unit length of stay (ICULOS), initiation of inpatient hospice, and Glasgow Outcome Scale (GOS) at discharge. Patients were divided based on whether their first renal replacement therapy after admission for traumatic injury was IHD or CRRT. Demographic and clinical data were presented as frequencies and percentages for categorical variables and as means and standard deviations for continuous variables. Continuous variables were compared using an independent t-test or Mann-Whitney U test as appropriate, and categorical variables were compared using chi-squared testing. SPSS version 27 (Armonk, NY: IBM Corp.) was used for analysis, and a p-value of ≤0.05 was used to determine statistical significance. Because this was a retrospective study utilizing all available eligible cases during the study period, no a priori sample size or power calculation was performed.

## Results

A total of 81 patients met the inclusion criteria, of which 52 initially received IHD, and 29 initially received CRRT during their ICU admission. Both groups had a higher proportion of male patients, and a larger proportion of patients in the CRRT group were male (79.3% vs. 57.7%; p=0.05). IHD group was associated with older age (68.81 vs. 58.41 years, p=0.006), and a higher average CCI (6.62 vs. 3.45, p<0.001). Patients were 1.81 times more likely to undergo IHD vs. CRRT for each additional one-point increase in CCI (95% CI: 1.20-2.71). CRRT use was associated with a significantly lower mean admission GCS than in the IHD group (8.69 vs. 13.52; p < 0.001). CRRT was also associated with ISS being higher (24.45 vs. 16.18; p=0.006) (Table 1).

**Table 1:** Demographic data and injury characteristics of included patients, stratified by type of dialysis initiated upon admission. Continuous variables were compared using an independent t-test or Mann-Whitney U test as appropriate, and categorical variables were compared using chi-squared testing. CRRT: continuous renal replacement therapy; IHD: intermittent hemodialysis

Patient characteristics	CRRT (n=29)	IHD (n=52)	p-Value
Age, mean years±standard deviation (SD)	58.4±18.4	68.8±14.2	0.006
Gender			
Male, n (%)	23 (79.3)	30 (57.7)	0.05
Female, n (%)	6 (20.7)	22 (42.3)	
Injury characteristics			
Admission Glasgow Coma Score, mean (SD)	8.7 (5.1)	13.5 (2.8)	<0.001
Injury severity score, mean (SD)	24.5 (8.3)	16.2 (6.7)	0.006
Charlson Comorbidity Index, mean (SD)	3.5 (2.6)	6.6 (2.4)	<0.001
Hemorrhage type, n (%)			
Epidural	2 (6.9)	1 (1.9)	0.29
Subdural	18 (62.1)	37 (71.2)	0.461
Intraventricular	4 (13.8)	6 (11.5)	0.74
Subarachnoid	19 (65.5)	25 (48.1)	0.131
Intraparenchymal	9 (31.0)	8 (15.4)	0.097
Other	1 (3.4)	0 (0.0)	0.358

There were no significant differences in the frequency of different intracranial bleeds. Namely, there was no association between use of CRRT vs. IHD with respect to incidence of subdural hematomas (62.1% vs. 71.2%, p=0.46), subarachnoid hemorrhage (65.5% vs. 48.1%, p=0.13), epidural hematomas (6.9% vs. 1.9%, p=0.32), intraventricular hemorrhage (13.8% vs. 11.5%, p=0.74), or intraparenchymal hemorrhage (31.0% vs. 15.4%, p=0.1). Subdural hematomas were the most common injury, accounting for 67.9% of the study population. Urgent neurosurgical intervention (including intracranial pressure monitor placement, craniotomy, burr hole craniotomy, or external ventricular drain catheter placement) was deemed necessary in 16 patients. Among those patients, there was no difference in the use of CRRT vs. IHD (24.1% vs. 18.4%, p=0.542). CRRT use was associated with significantly higher mortality (62.1% vs. 30.8%, p<0.001), a longer ICULOS (13 vs. two days, p<0.001), and a longer hospital LOS (HLOS) (17 vs. 8.6 days, p=0.001), with no difference in rates of transfer to inpatient hospice (13.8% vs. 15.7%, p=1). In terms of Glasgow Outcome Scale (GOS) at discharge, the CRRT group had more patients discharged with severe disability (17.2% vs. 3.8%, p=0.001), and fewer patients discharged with moderate (10.3% vs. 25.0%, p=0.001) or low disability (10.3% vs. 40.4%, p=0.001) (Table 2).

**Table 2:** Outcomes of included patients, stratified by type of dialysis initiated. Continuous variables were compared using an independent t-test or Mann-Whitney U test as appropriate, and categorical variables were compared using chi-squared testing. CRRT: continuous renal replacement therapy; IHD: intermittent hemodialysis

Outcomes	CRRT (n=29)	IHD (n=52)	p-Value
Urgent surgery needed, n (%)	7 (24.1)	9 (18.4)	0.542
Inpatient hospice, n (%)	4 (13.8)	8 (15.7)	1
Glasgow Outcome Scale at discharge			
Death, n (%)	18 (62.1)	16 (30.8)	<0.001
Severe disability, n (%)	5 (17.2)	2 (3.8)	
Moderate disability, n (%)	3 (10.3)	13 (25.0)	
Low disability, n (%)	3 (10.3)	21 (40.4)	
Intensive care length of stay, median days (IQR)	13 (7.5-27.5)	2 (0-5)	<0.001
Hospital length of stay, median days (IQR)	17 (8.3-45.1)	7 (3.5-14.0)	<0.001

## Discussion

While most literature has advocated for CRRT in the context of TBI to prevent increased intracranial pressure and the risk for dialysis disequilibrium syndrome (DDS) and ultimately herniation [10,13,14,16,18,20], Martinez-Gonzalez et al. demonstrated the opposite [21]. In an animal model, CRRT worsened the severity of acute traumatic brain injury [21]. The literature also does not agree on the specific benefits and the extent of these benefits conferred on TBI patients initiated on CRRT vs. IHD. The only randomized controlled trial evaluating these methods of renal replacement in patients with pre-existing renal dysfunction complicated by intracerebral hemorrhage, a condition comparable to acute TBI, demonstrated that the CRRT group had less cerebral edema, fewer adverse events, and shorter recoveries than the IHD group. However, the applicability of this study to trauma patients is debatable [22]. In patients with traumatic injuries, studies have largely failed to demonstrate a mortality benefit from CRRT over IHD [6,23]; however, Ghahramani et al. found that patients treated with CRRT demonstrated a greater degree of renal recovery and less ultimate dialysis dependence [6]. Schoenfelder et al. also found that despite initiation of CRRT, patients sustained higher mortality upfront compared to IHD patients, and those surviving CRRT were less likely to experience renal recovery [24]. Similar to our study, the higher mortality rate associated with the use of CRRT was likely a result of a higher burden of injuries, biasing providers towards selecting CRRT initiation over IHD.

It is evident that renal dysfunction in the setting of TBI is a complex and dynamic process known to increase mortality in trauma patients [4]. Some have argued that severe TBIs potentiate acute kidney injury (AKI) [3], which peaks in incidence in the first three days after a TBI [4], yet it is also known that AKI worsens TBI [25]. Although several recent studies have evaluated the epidemiology and management of patients with TBI who develop AKI, the majority of existing literature has been anecdotal, theoretical, or case series of fewer than ten patients. Furthermore, there is limited published literature specifically examining acute traumatic brain injury patients with pre-existing chronic kidney disease.

**Figure 1:**
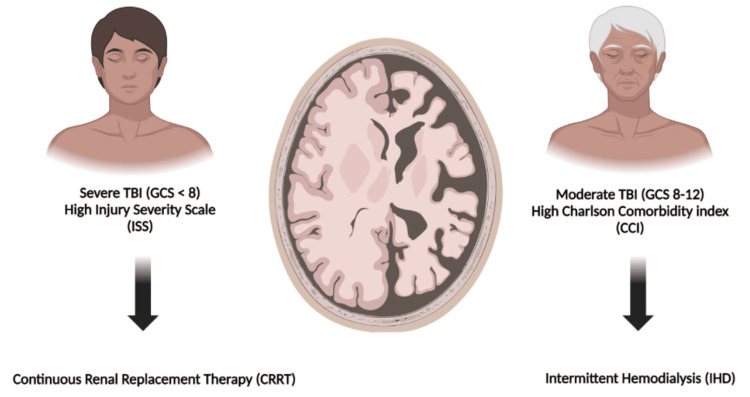
Dialysis modality selection in TBI. This image was created by Michael Bamimore using Biorender.com. TBI: traumatic brain injury GCS: Glasgow Coma Scale

Our finding that older age was associated with IHD receipt is noteworthy. Physiologic cerebral atrophy in older patients decreases intracranial parenchymal volume, allowing for decreased incidence of DDS. Perhaps a younger population with a lower CCI but worsened intracranial pathology is at higher risk for DDS, given the lack of ability to volumetrically expand. Thus, age-specific responses to CRRT or IHD should be investigated in future trials. Figure 1 further illustrates the typical clinical and radiographic scenarios in which we favor CRRT over IHD in the setting of TBI with concomitant AKI at our institution.

Our study is consistent with prior literature documenting a low incidence of patients sustaining TBI and requiring renal replacement therapy, which has likely prohibited high-powered analysis of this patient population [2,5,6,17,23]. Few studies have systematically evaluated the use of continuous renal replacement therapy in patients with traumatic brain injury requiring dialysis [17]. However, the documented mortality was exceedingly high, and only 24-h urine output before CRRT initiation was noted on multivariate analysis to be predictive of mortality [17]. CRRT use was also not directly compared to IHD use; patients who received IHD upfront were excluded from the analysis [17]. Additionally, given the low prevalence of pre-existing chronic kidney disease and the need for renal replacement therapy in this population, this study was underpowered to demonstrate a big difference.

​There are several limitations to our study. As a single-center, retrospective review, it is inherently biased. Given the descriptive nature of this research, causality cannot be inferred. The small size of our cohort from a single institution prohibits these observations from being broadly generalizable. The sample size limitation prevented multivariable adjustment for factors such as age, injury severity score (ISS), GCS, or comorbidity burden, which limits the ability to discern whether observed differences between CRRT and IHD reflect independent effects of modality rather than underlying differences in illness severity. Additionally, patient outcomes were assessed using an intention-to-treat model, in which patients who initially received IHD or CRRT were compared, regardless of whether a patient's method of renal replacement was changed from one of these methods to the other during the admission. Furthermore, this study was unable to evaluate patients with pre-existing CKD based on the available data; however, it attempted to capture this by using the CCI to assess each patient’s burden of pre-existing health conditions. Only two other single-center studies have evaluated this patient population, suggesting that despite these limitations, our study contributes to the body of available data. Age-specific data or patient matching was not conducted, given the nature of the study, further limiting its applicability. The nuances of injury type and additional patient characteristics would be better demonstrated in a larger cohort study, as the higher mortality observed in the CRRT group was likely driven by higher baseline illness severity rather than by the dialysis modality itself.

Despite these shortcomings, our study provides valuable insight into physician decision-making in the management of patients with TBI requiring renal replacement therapy. At our institution, the decision to use CRRT rather than IHD in patients with TBI was most strongly associated with each patient’s admission GCS and ISS, favoring CRRT among more severely injured patients. Patients’ pre-existing comorbid conditions seemed to have minimal impact on this decision, as evidenced by the fact that patients with higher CCI scores more often received IHD, when the opposite was hypothesized to be true. Kidney injury in the context of TBI is a significant source of morbidity and mortality for trauma patients, and management of coexisting conditions, such as kidney injury, should be studied further.

## Conclusions

Patients sustaining TBI and concomitantly requiring dialysis represent a small but critically ill cohort. In this study, lower admission GCS and CCI, and higher ISS were associated with the use of CRRT rather than IHD. While these associations may offer context for clinicians weighing the risks of dialysis disequilibrium syndrome against comorbidity burden and injury severity, they should not be interpreted as causal for the outcomes assessed. Instead, understanding these patterns of practice may inform the development of a more thoughtful protocol to optimize both neurologic and renal outcomes in severely injured patients. Further investigation is needed to establish evidence-based management strategies for this vulnerable population.
